# Elevation of Hyaluronan Synthase by Magnesium Supplementation Mediated through the Activation of GSK3 and CREB in Human Keratinocyte-Derived HaCaT Cells

**DOI:** 10.3390/ijms23010071

**Published:** 2021-12-22

**Authors:** Kana Marunaka, Shokoku Shu, Mao Kobayashi, Makiko Goto, Yuji Katsuta, Yuta Yoshino, Akira Ikari

**Affiliations:** 1 Laboratory of Biochemistry, Department of Biopharmaceutical Sciences, Gifu Pharmaceutical University, Gifu 501-1196, Japan; 136033@gifu-pu.ac.jp (K.M.); 165041@gifu-pu.ac.jp (S.S.); 155032@gifu-pu.ac.jp (M.K.); yoshino-yu@gifu-pu.ac.jp (Y.Y.); 2 MIRAI Technology Institute, Shiseido Co. Ltd., Kanagawa 220-0011, Japan; makiko.goto@shiseido.com (M.G.); yuji.katsuta@shiseido.com (Y.K.)

**Keywords:** hyaluronic acid, hyaluronan synthase, magnesium

## Abstract

Skin barrier damage is present in the patients with hereditary disorders of the magnesium channel, but the molecular mechanism has not been fully understood. We found that the expressions of hyaluronan synthase (HAS), HAS2 and HAS3 are influenced by MgCl_2_ concentration in human keratinocyte-derived HaCaT cells. The exposure of cells to a high concentration (5.8 mM) of MgCl_2_ induced the elevation of HAS2/3 expression, which was inhibited by mRNA knockdown of nonimprinted in Prader-Willi/Angelman syndrome-like domain containing 4 (NIPAL4). Similarly, the content of hyaluronic acid (HA) was changed according to MgCl_2_ concentration and the expression of NIPAL4. The MgCl_2_ supplementation increased the reporter activities of HAS2/3, which were inhibited by NIPAL4 knockdown, indicating that the expressions of HAS2/3 are up-regulated at the transcriptional level. The reporter activities and mRNA levels of HAS2/3, and the production of HA were inhibited by CHIR-99021, a glycogen synthase kinase-3 (GSK3) inhibitor, and naphthol AS-E, a cyclic AMP-response element binding protein (CREB) inhibitor. Furthermore, the mutation in putative CREB-binding sites of promoter region in HAS2/3 genes inhibited the MgCl_2_ supplementation-induced elevation of promoter activity. Our results indicate that the expressions of HAS2/3 are up-regulated by MgCl_2_ supplementation in HaCaT cells mediated through the activation of GSK3 and CREB. Magnesium may play a pivotal role in maintaining the skin barrier function and magnesium supplementation may be useful to enhance moisturization and wound repair in the skin.

## 1. Introduction

Autosomal recessive congenital ichthyosis (ARCI) is a debilitating skin disease characterized by aberrant barrier function and diffuse skin scaling. ARCI can be clinically divided into three types, including harlequin ichthyosis (HI), lamellar ichthyosis (LI), and congenital ichthyosiform erythroderma (CIE) [1]. HI is the most phenotypically severe ARCI associated with the mutation of the adenosine triphosphate (ATP) binding cassette subfamily A member 12 (ABCA12) gene. On the other hand, mutations in various genes including ABCA12, transglutaminase 1 (TGM1), nonimprinted in Prader-Willi/Angelman syndrome-like domain-containing 4 (NIPAL4), 3-lipoxygenase (ALOXE3), 12-lipoxygenase (ALOX12B), CYP4F22, and loci on 12p11.2-q13 are implicated in the pathogenesis of LI and CIE.

NIPAL4, also known as ichthyin, codes for putative magnesium transporter NIPA4, which is composed of several transmembrane domains. In situ hybridization shows that NIPA4 is expressed in the epidermis and localized in the granular layer [2]. NIPAL4-knockout (KO) mice exhibit neonatal lethality due to skin barrier defects [3]. The Mg^2+^ concentration in differentiated keratinocytes in NIPAL4-KO mice is lower than that in wild-type mice. Magnesium is a divalent cation most abundantly existing in the cells of the human body. Magnesium plays a pivotal role in more than 300 enzymatic reactions and is involved in the regulation of physiological roles including glucose metabolization, protein synthesis, energy production, and so on [4]. As mentioned above, the patients with ARCI have mutations in the NIPAL4 gene, indicating NIPA4 may be involved in the maintenance of magnesium homeostasis and skin barrier integrity. However, the function of magnesium in the skin remains unclear.

The function of skin is to maintain body hydration, temperature, moisture, and sensations [5]. Hyaluronic acid (HA), consisting of alternating *N*-acetylglucosamine and glucuronic acid units, is a most general component of the extracellular matrix in the vertebrate and plays crucial roles in skin moisture and elasticity [6]. Approximately one-third of the body’s total HA is turned over daily, which is controlled by synthesis and degradation processes. HA synthases are membrane-bound enzymes and divided into three isoforms, HAS1, HAS2 and HAS3 [7]. Three HAS isoforms share a high level of homology (55–71%) [8] and HAS2 is most abundantly expressed in keratinocytes. Epidermal growth factor (EGF) and all-trans-retinoic acid (RA) accelerate wound-healing mediated through a HAS2-dependent HA production in skin diseases [9].

The expression patterns of the HAS isoforms differ in the tissues of humans. Furthermore, the expression of HAS isoforms and HA production are up- or down-regulated under various pathophysiological conditions including tissue injury, inflammation, and cancer. Experimental data and in silico screening reveal that the promoter region of each HAS gene contains various binding motifs of transcription factors including cAMP-responsive element binding proteins (CREB), nuclear factor-kappa B (NF-κB), Sp1, and E2F [10]. The expression of HAS2 is regulated by some natural products. HAS2 expression and HA production in human keratinocyte-derived HaCaT cells are up-regulated by Kahweol, a food factor contained in coffee [11], 3,6-anhydro-l-galactose, a main component of red macroalgal carbohydrates [12], and epigallocatechin gallate, a catechin contained in green tea [13]. Seed oil of sea buckthorn increases HAS2 expression in normal human epidermal keratinocytes cells [14]. On the other hand, it is unknown whether Mg^2+^ can affect HAS expression and HA production.

In the present study, we investigated the effects of MgCl_2_ supplementation and depletion on HAS expression using HaCaT cells. The mRNA and protein levels were examined using real-time polymerase chain reaction (PCR) and Western blotting analyses, respectively. Intracellular free Mg^2+^ concentration ([Mg^2+^]_i_) was measured using KMG-20, a Mg^2+^-sensitive fluorescent dye. The reporter activities of HAS2/3 were assessed by luciferase assay. Our results indicate that Mg^2+^ may play a pivotal role in the production of HA.

## 2. Results

### 2.1. Effect of Extracellular MgCl_2_ Concentration on Expression of HAS2/3

**Figure 1:**
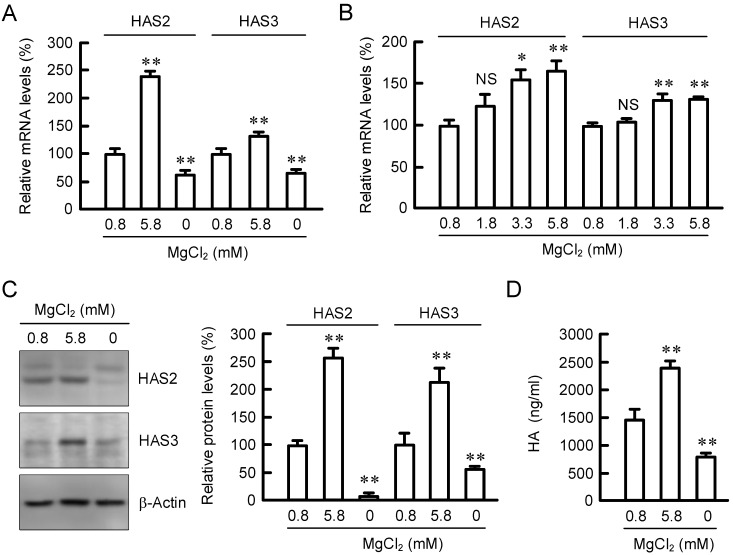
Effects of extracellular Mg^2+^ concentration on HAS2/3 expressions in HaCaT cells. (**A**,**B**) Cells were incubated in the presence of 0, 0.8, and 5.8 mM MgCl_2_ for 6 h. The mRNA levels of HAS2/3 were measured by real-time PCR analysis and represented as a percentage of 0.8 mM MgCl_2_. (**C**,**D**) Cells were incubated in the presence of 0, 0.8, and 5.8 mM MgCl_2_ for 24 h. The protein levels of HAS2/3 were measured by Western blotting analysis and represented as a percentage of 0.8 mM MgCl_2_. HA contents in the media were measured using a Hyaluronan Quantification kit. *n* = 3–4. ** *p* < 0.01 and * *p* < 0.05 significantly different from 0.8 mM MgCl_2_. ^NS^ *p* > 0.05.

HaCaT cells were exposed to the media containing 0.8 mM MgCl_2_ (Normal), 5.8 mM MgCl_2_ (High), or 0 mM MgCl_2_ (Nominally free) for 6 h. The mRNA levels of HAS2/3 were significantly increased in 5.8 mM MgCl_2_ medium compared with 0.8 mM MgCl_2_ medium, whereas they were decreased in 0 mM MgCl_2_ medium (Figure 1A). In contrast, the mRNA level of HAS1 was below the detection limit. The mRNA levels of HAS2/3 were increased by MgCl_2_ supplementation in a dose-dependent manner (Figure 1B). The protein levels of HAS2/3 and HA production were changed depending on extracellular MgCl_2_ concentration (Figure 1C,D), which coincide with the results of mRNA. In addition, the elevation of HAS2/3 mRNAs was observed by the supplementation of magnesium lactate or magnesium sulfate (Supplementary Figure S1). These results indicate that Mg^2+^ concentration is involved in the regulation of HAS2/3 expression and HA production in HaCaT cells.

### 2.2. Effect of NIPAL4 siRNA on the Expressions of HAS2/3

**Figure 2:**
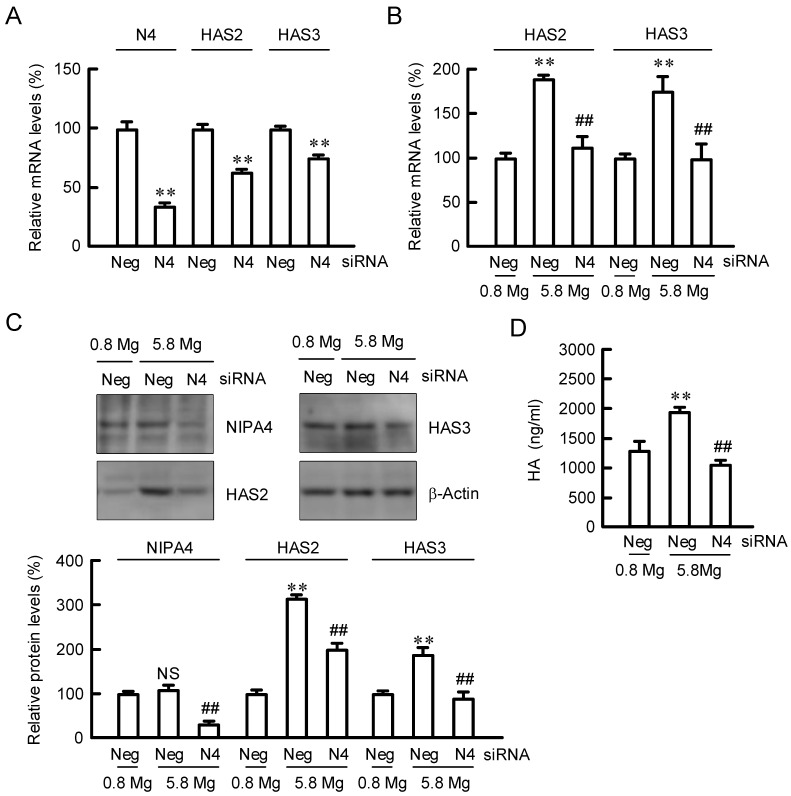
Effect of NIPAL4 siRNA on HAS2/3 expressions. Cells were transfected with negative (Neg) or NIAPL4 (N4) siRNA. (**A**) After transfection, the cells were incubated in the presence of 0.8 mM MgCl_2_ for 72 h. The mRNA levels of N4 and HAS2/3 were measured by real-time PCR analysis and represented as a percentage of negative siRNA. (**B**) After 66 h of transfection, the cells were incubated in the presence of 0.8 and 5.8 mM MgCl_2_ for 6 h. The mRNA levels of HAS2/3 were measured by real-time PCR analysis and represented as a percentage of 0.8 mM MgCl_2_. (**C**,**D**) After 48 h of transfection, the cells were incubated in the presence of 0.8 and 5.8 mM MgCl_2_ for 24 h. The protein levels of NIPA4 and HAS2/3 were measured by Western blotting analysis and represented as a percentage of 0.8 mM MgCl_2_. HA contents in the media were measured using a Hyaluronan Quantification kit. *n* = 3–4. ** *p* < 0.01 significantly different from negative siRNA or 0.8 mM MgCl_2_. ^NS^ *p* > 0.05. ^##^ *p* < 0.01 significantly different from negative siRNA plus 5.8 mM MgCl_2_.

The mRNA level of NIPAL4 was significantly decreased by the introduction of its siRNA, (Figure 2A). In addition, the mRNA levels of HAS2/3 were decreased by NIPAL4 knockdown. The MgCl_2_ supplementation-induced elevation of HAS2/3 mRNAs was inhibited by NIPAL4 knockdown (Figure 2B). Similarly, the protein levels of HAS2/3 and HA production were increased by MgCl_2_ supplementation, which were inhibited by NIPAL4 knockdown (Figure 2C). These results indicate that Mg^2+^ influx mediated through NIPAL4 may be involved in the elevation of HAS2/3 by MgCl_2_ supplementation.

### 2.3. Effects of MgCl_2_ Supplementation and NIPAL4 siRNA on [Mg^2+^]_i_

**Figure 3:**
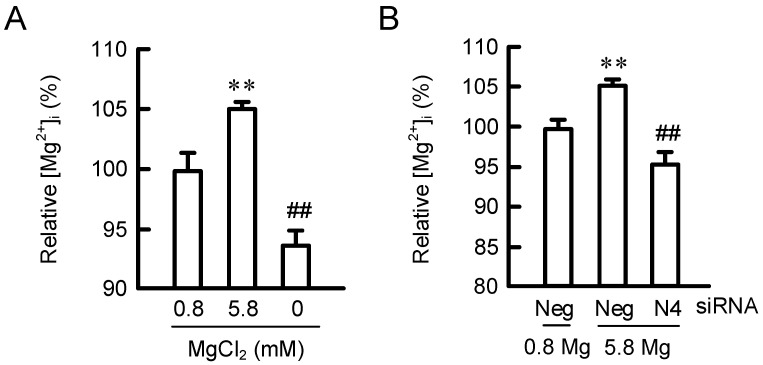
Effects of extracellular Mg^2+^ concentration and NIPAL4 siRNA on [Mg^2+^]_i_. (**A**) Cells were incubated in the presence of 0, 0.8, and 5.8 mM MgCl_2_ for 24 h. The cells were loaded with KMG-20-AM for 30 min at 37 °C. The fluorescence intensity of KMG-20 was measured using a plate reader. [Mg^2+^]_i_ is represented as a percentage of 0.8 mM MgCl_2_. (**B**) Cells were transfected with negative (Neg) or NIPAL4 (N4) siRNA. After 48 h of transfection, the cells were incubated in the presence of 0.8 and 5.8 mM MgCl_2_ for 24 h. [Mg^2+^]_i_ is represented as a percentage of 0.8 mM MgCl_2_. *n* = 4–6. ** *p* < 0.01 significantly different from 0.8 mM MgCl_2_. ^##^ *p* < 0.01 significantly different from negative siRNA plus 5.8 mM MgCl_2_.

The [Mg^2+^]_i_ in HaCaT cells was monitored using a fluorescent Mg^2+^ indicator, KMG-20. The fluorescence intensity was increased by exposing to 5.8 mM MgCl_2_ medium, whereas that was decreased by the 0 mM MgCl_2_ medium (Figure 3). The MgCl_2_ supplementation-induced elevation of fluorescence intensity was significantly inhibited by NIPAL4 knockdown. These results indicate that NIPA4 may function as Mg^2+^ channel in HaCaT cells.

### 2.4. Effect of MgCl_2_ Supplementation and NIPAL4 siRNA on Reporter Activities of HAS2/3

**Figure 4:**
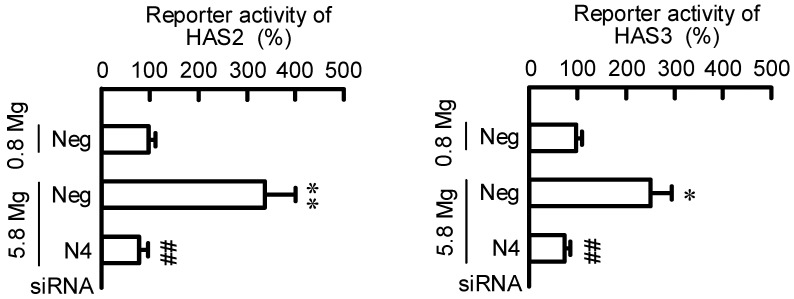
Effects of extracellular Mg^2+^ concentration and NIPAL4 siRNA on reporter activities of HAS2/3. Cells were transfected with negative (Neg) or NIAPL4 (N4) siRNA. After 66 h of transfection, the cells were incubated in the presence of 0.8 and 5.8 mM MgCl_2_ for 6 h. The activities of secreted luciferase and SEAP were measured using a Ready-To-Glow Dual Secreted Reporter Assay kit. The reporter activity is represented as a percentage of 0.8 mM MgCl_2_. *n* = 4. ** *p* < 0.01 and * *p* < 0.05 significantly different from 0.8 mM MgCl_2_. ^##^ *p* < 0.01 significantly different from negative siRNA plus 5.8 mM MgCl_2_.

HaCaT cells were transiently transfected with HAS2 or HAS3 reporter plasmid plus an internal control plasmid. The reporter activities of HAS2/3 were increased in 5.8 mM MgCl_2_ medium compared with that in 0.8 mM MgCl_2_ medium, which was inhibited by NIPAL4 knockdown (Figure 4). These results are similar to those in the mRNA and protein levels of HAS2/3, indicating the expressions of HAS2/3 may be regulated at the transcriptional step by MgCl_2_ concentration.

### 2.5. Effects of MgCl_2_ Supplementation and NIPAL4 siRNA on Cell Migration

**Figure 5:**
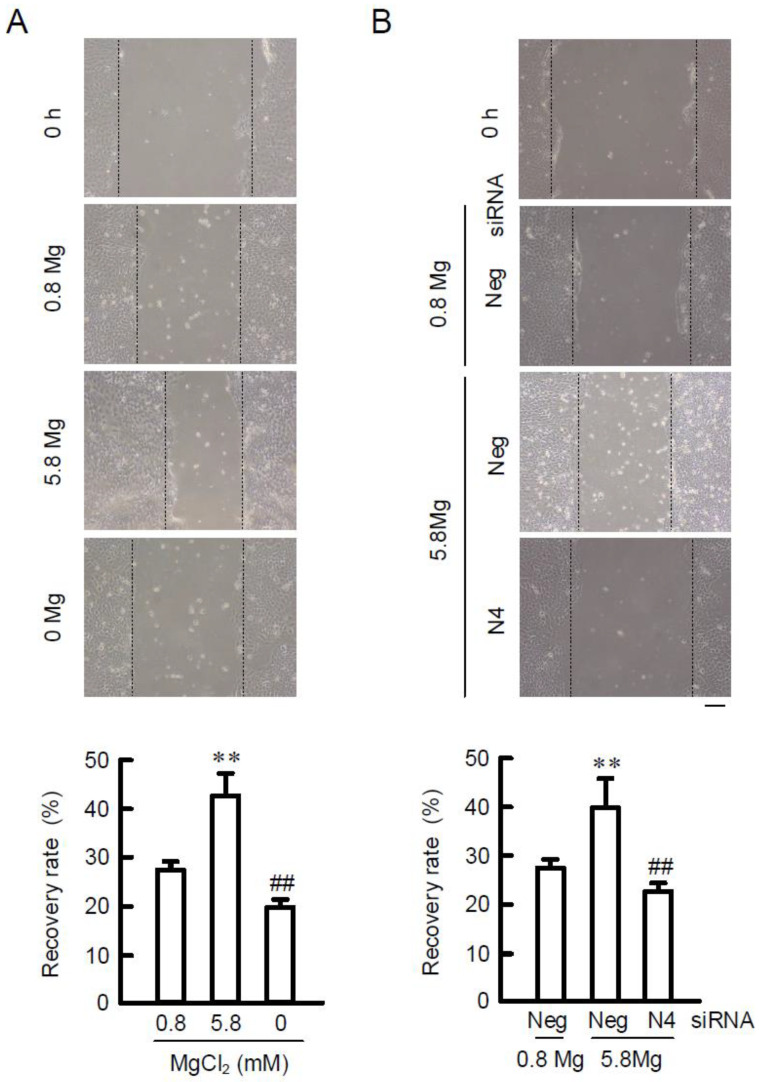
Effects of extracellular Mg^2+^ concentration and NIPAL4 siRNA on cell migration. (**A**) After reaching confluent densities, the cells were incubated in 0, 0.8, and 5.8 mM MgCl_2_ media for 24 h. Then, the cells were scratched with tip of a 200-μL pipette tip. The image of 0 h was taken at just after scratch. Other images were taken after 24 h. (**B**) Cells were transfected with negative (Neg) or NIPAL4 (N4) siRNA. After 66 h of transfection, the cells were incubated in 0.8 and 5.8 mM MgCl_2_ media for 24 h. Then, the cells were scratched with tip of a 200-μL pipette tip. The images were taken after 24 h. The recovery rate is presented as the percentage of scratch closure. Scale bar indicates 50 μm. *n* = 4–6. ** *p* < 0.01 significantly different from 0.8 mM MgCl_2_. ^##^ *p* < 0.01 significantly different from 5.8 mM MgCl_2_ or negative siRNA plus 5.8 mM MgCl_2_.

HA facilitates cell migration and wound-healing in the skin [15]. In the wound-healing assay, the recovery rate was increased in 5.8 mM MgCl_2_ medium compared with that in 0.8 mM MgCl_2_ medium, whereas that was decreased in 0 mM MgCl_2_ medium (Figure 5). The MgCl_2_ supplementation-induced elevation of recovery rate was inhibited by NIPAL4 knockdown. These results are similar to those in HAS2/3 expressions and HA production.

### 2.6. Effect of MgCl_2_ Supplementation on Intracellular Signaling Pathways

**Figure 6:**
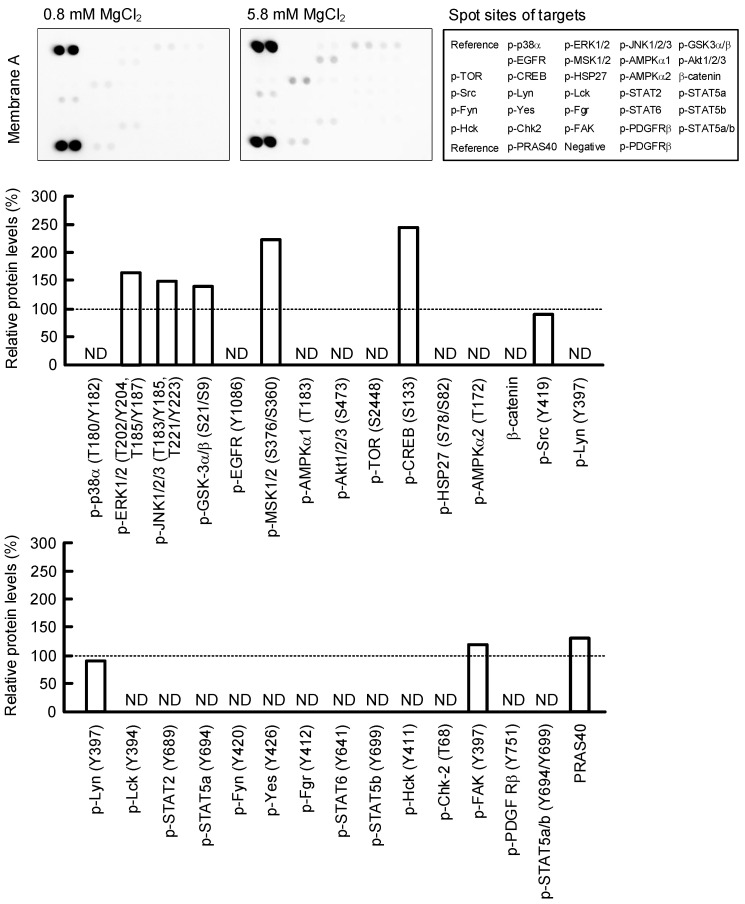

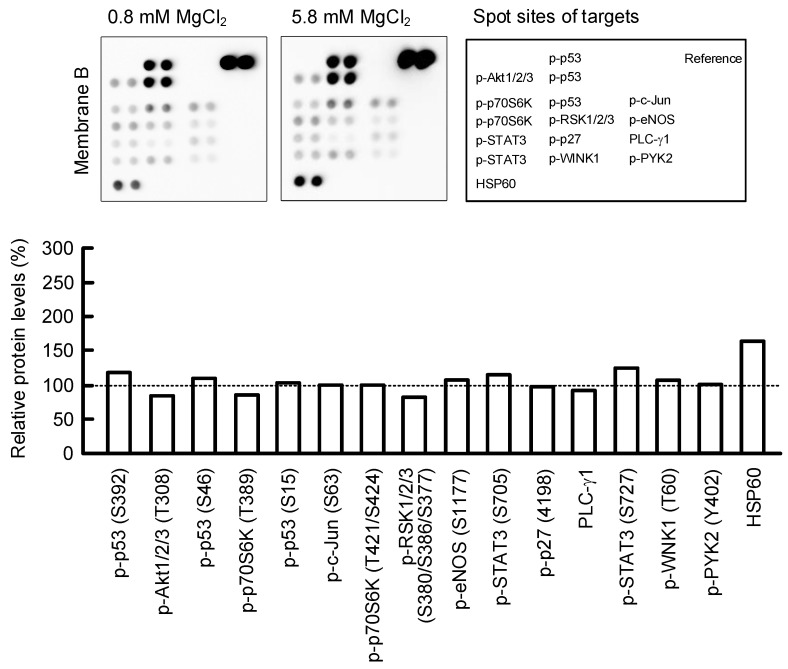
Effect of MgCl_2_ supplementation on phosphorylation of intracellular signaling proteins. Cells were incubated in 0.8 and 5.8 mM MgCl_2_ media for 6 h. After collecting whole cell lysates, the aliquots were applied on a Proteome Profiler Human Phospho-Kinase Array Kit. **Upper images** indicate the membrane A and B, and spot sites of targets. **Lower images** indicate the phosphorylation level or total amount are represented as a percentage of 0.8 mM MgCl_2_. A signal below the detection limit is indicated by non-detectable (ND).

**Figure 7:**
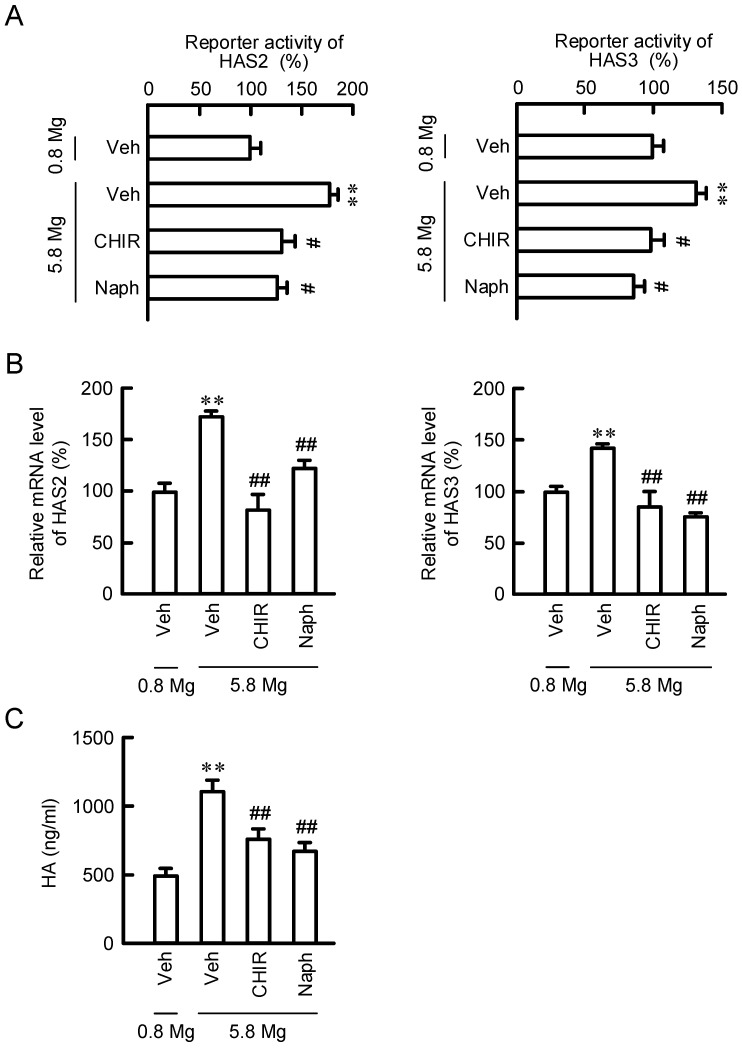
Effects of MgCl_2_ supplementation and signaling inhibitors on HAS2/3 expressions and HA production. (**A**) Cells transfected with promoter reporter vectors for HAS2/3 were incubated in the absence (Veh) and presence of 10 μM CHIR-99021 (CHIR) or 10 μM naphthol AS-E (Naph) for 6 h. The reporter activities of HAS2/3 are represented as a percentage of 0.8 mM MgCl_2_. (**B**) Cells were incubated in the absence (Veh) and presence of 0.8 mM MgCl_2_, 5.8 mM MgCl_2_, 10 μM CHIR-99021 (CHIR) or 10 μM naphthol AS-E (Naph) for 6 h. The mRNA levels of HAS2/3 are represented as a percentage of 0.8 mM MgCl_2_. (**C**) Cells were incubated in the absence (Veh) and presence of 0.8 mM MgCl_2_, 5.8 mM MgCl_2_, 10 μM CHIR-99021 (CHIR) or 10 μM naphthol AS-E (Naph) for 24 h. After collecting the media, HA contents were measured using a Hyaluronan Quantification kit. *n* = 4–6. ** *p* < 0.01 significantly different from 0.8 mM MgCl_2_. ^##^ *p* < 0.01 and ^#^ *p* < 0.05 significantly different from 5.8 mM MgCl_2_ plus 5.8 mM MgCl_2_.

To clarify the regulatory mechanisms of HAS2/3 expressions, the phosphorylation levels of intracellular signaling proteins were investigated using a Proteome Profiler Human Phospho-Kinase Array Kit. The protein levels of p-ERK1/2, p-JNK1/2/3, p-GSK3α/β, p-MSK1/2, p-CREB, p-FAK, PRAS40, p-STAT3, and HSP60 were increased over 1.2-fold in 5.8 mM MgCl_2_ medium compared with 0.8 mM MgCl_2_ medium (Figure 6). Other protein levels were constant or below the detection limit. Recently, Terazawa et al. [16] reported that mycosporine-like amino acids increase HAS2 expression in human dermal fibroblasts mediated by up-regulating the activation of the p38/CREB/AP-1 pathway. The level of p-p38 was below the detection limit and that of p-c-Jun, a component of AP-1, was not increased in 5.8 mM MgCl_2_ medium in HaCaT cells. Therefore, we eliminated the involvement of p38 and AP-1. The phosphorylation of CREB is up-regulated by not only p38, but also GSK3α/β [17]. Therefore, we investigated the effects of specific inhibitors against GSK3α/β and CREB. The MgCl_2_ supplementation increased the reporter activities, mRNA levels of HAS2/3, and HA production in HaCaT cells, which were inhibited by CHIR-99021, GSK3α/β inhibitor, and naphthol AS-E, a CREB inhibitor (Figure 7). These results indicate that the activation of GSK3α/β and CREB may be involved in the MgCl_2_ supplementation-induced elevation of HAS2/3 expressions.

### 2.7. Regulation of Transcription Activities of HAS2/3 by CREB

**Figure 8:**
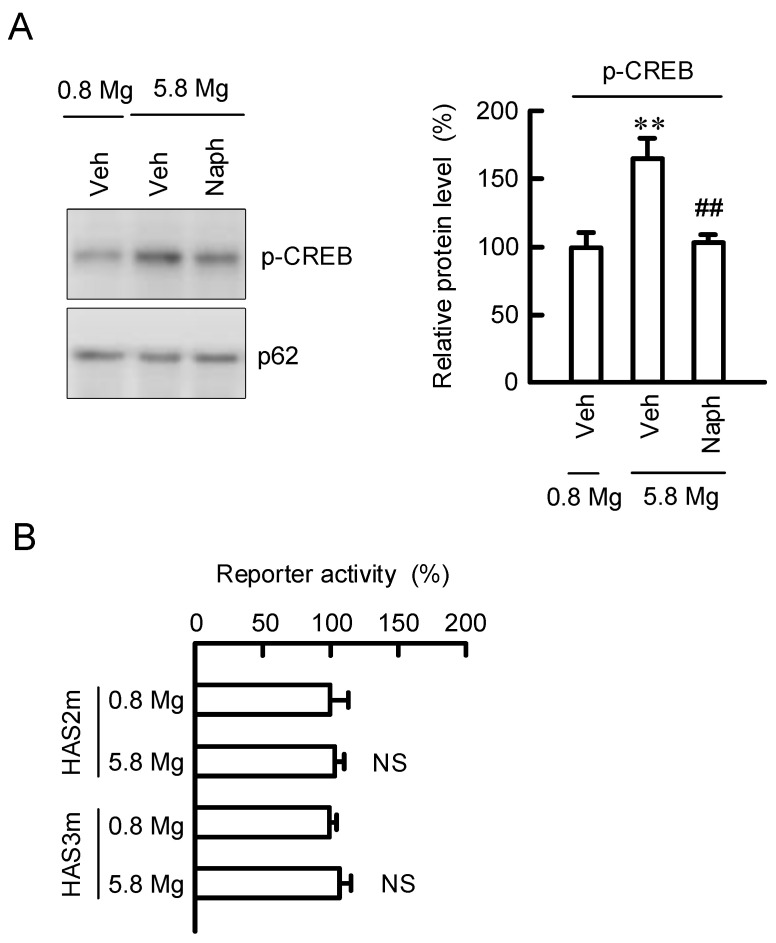
Involvement of CREB in the elevation of HAS2/3 expressions by MgCl_2_ supplementation. (**A**) Cells were incubated in the absence (Veh) and presence of 0.8 mM MgCl_2_, 5.8 mM MgCl_2_, or 10 μM naphthol AS-E (Naph) for 2 h. After isolation of nuclear fraction, the protein levels of p-CREB and nucleoporin p62 (p62), an internal control, were measured by Western blotting analysis and represented as a percentage of 0.8 mM MgCl_2_. (**B**) Cells were transfected with reporter vectors of HAS2/3 containing the mutation in CREB-binding site (HAS2m and HAS3m). Then, the cells were incubated in the presence of 0.8 and 5.8 mM MgCl_2_ for 6 h. The reporter activity is represented as a percentage of 0.8 mM MgCl_2_. *n* = 3–4. ** *p* < 0.01 significantly different from 0.8 mM MgCl_2_. ^NS^ *p* > 0.05. ^##^ *p* < 0.01 significantly different from 5.8 mM MgCl_2_ plus 5.8 mM MgCl_2_.

The nuclear contents of p-CREB were examined by Western blotting analysis. The MgCl_2_ supplementation increased the contents of p-CREB in the nuclear fraction, which was inhibited by naphthol AS-E (Figure 8A). The transcription factor prediction program TFSEARCH showed that the promoter regions of HAS2 and HAS3 contain a putative CREB-binding site. The elevation of promoter activities of HAS2/3 caused by MgCl_2_ supplementation was inhibited by the mutation in the CREB-binding site of each promoter region (Figure 8B). These results indicate that MgCl_2_ supplementation may increase HAS2/3 expressions mediated by the nuclear localization and binding of CREB to promoter region of HAS2/3 genes.

## 3. Discussion

The epidermis of NIPAL4-KO mice shows several morphological abnormalities including impairment of lipid multilayer structure formation, hyperkeratosis, immature keratohyalin granules, and developed heterochromatin structures [3]. A reduction of Mg^2+^ concentration in differentiated keratinocytes is observed in NIPAL4-KO mice. Our results indicate that the basal [Mg^2+^]_i_ in the HaCaT cells transfected with NIPAL4 siRNA was lower than that with negative siRNA (Figure 3). Electrophysiological study revealed that NIPA4 can transport Mg^2+^ in Xenopus oocytes [18]. NIPA4 may play a pivotal role in the regulation of basal [Mg^2+^]_i_ in the epidermis. The patients with NIPAL4 mutation show increased 12R-lipoxygenase (LOX) and epidermal LOX-3 staining, which are over three times than normal epidermis [19], indicating that abnormal lipid accumulation may be associated with the dysfunction of the skin barrier. However, the role of Mg^2+^ in the maintenance of skin-barrier homeostasis remains unknown.

HA is the most common glycosaminoglycan of the extracellular matrix and used dermal filler. HA is synthesized by specific enzymes of HAS1–3, and among them HAS2 is shown to be the predominant isoform in the skin dermis [20]. The HAS2 expression is up-regulated by cyclic phosphatidic acid and lysophosphatidic acid in human skin fibroblasts [21], and EGF and RA in epidermal keratinocytes [9]. We found that HA production is increased by the MgCl_2_ supplementation in HaCaT cells (Figure 4 and Figure 8). HA is synthesized as a large polymer, high molecular weight HA (>250 kDa), and then it can be degraded into low molecular weight HA (LMW-HA) fragments by the activity of hyaluronidases or reactive oxygen species [22]. LMW-HA enhances not only extracellular matrix remodeling, but also wound repair in the skin [23,24]. The recovery rate of the wound area was accelerated by preculturing in 5.8 mM MgCl_2_ medium, whereas that was decelerated in 0 mM MgCl_2_ medium (Figure 5). In addition, the high concentration of MgCl_2_-induced elevation of recovery rate was inhibited by NIPA4 knockdown. These results suggest that the addition of MgCl_2_ may enhance wound-healing mediated though the production of HA in keratinocytes. Denda et al. [25] reported that magnesium salt accelerates skin barrier recovery using the flank skin of hairless mice. Although it is unknown whether the high concentration of MgCl_2_ can increase the amount of LMW-HA, the application of MgCl_2_ may be useful to production of HA and promotion of skin barrier recovery.

The promoter region of the HAS2 gene contains functional response elements for a lot of transcriptional factors including STAT3, CREB, SP1, and NF-κB [21]. The nuclear p-CREB level was increased by the MgCl_2_ supplementation, which was significantly inhibited by a CREB inhibitor (Figure 8A), suggesting that the high concentration of MgCl_2_ increases phosphorylation and translocation of CREB into the nuclei. In addition, the reporter assay (Figure 7A), real time PCR analysis (Figure 7B), and mutation analysis of HAS2/3 (Figure 8B) revealed that the MgCl_2_ supplementation-induced responses are inhibited by a CREB inhibitor. These results support the idea that high concentrations of MgCl_2_ may increase the expression of HAS2/3 mediated through the activation of CREB in the keratinocytes. Several intracellular signaling factors are involved in the up-regulation of CREB. Recently, Terazawa et al. [16] reported that mycosporine-like amino acids increase HAS2 expression in human dermal fibroblasts mediated by up-regulating the activation of p38/CREB/AP-1 pathway. In contrast, our data indicate that the levels of p-MSK1/2 and p-CREB were increased by 5.8 mM MgCl_2_, but that of c-Jun, a component of AP-1, was not. In addition, the MgCl_2_ supplementation-induced elevation of reporter activities of HAS2/3 were inhibited by mutation in CREB-binding regions. Therefore, we suggest that the MgCl_2_ supplementation increases the expression levels of HAS2/3 mediated through the activation of MSK1/GSK3/CREB pathway in the keratinocyte. The activation of the GSK3β signaling pathway may have a positive role in promoting epidermal differentiate in psoriasis [26]. In contrast, GSK3β may be involved in the fibroblast growth factor 19-induced hyperproliferation of keratinocytes in skin lesions [27]. The function of GSK3 must need to be evaluated carefully. Inhibitors of phosphodiesterase-4, which can activate cAMP-dependent CREB signaling, have been clinically used in the treatment of inflammatory diseases [28]. Our data indicate that the MgCl_2_ supplementation activates the CREB signaling in HaCaT cells. Animal experiments and clinical observation indicate the correlation between magnesium concentration and skin allergy reactions [29]. We suggest that the MgCl_2_ supplementation may be effective against not only HA production, but also immune suppression. Further studies are needed to clarify the clinical significance of MgCl_2_ supplementation.

In conclusion, we found that the expression of HAS2/3 and production of HA are regulated by extracellular Mg^2+^ concentration in HaCaT cells. The knockdown experiments using NIPAL4 siRNA suggested that these proteins function as the Mg^2+^ channel. Both HAS2/3 expression and HA production were in parallel changed by extracellular Mg^2+^ concentration. The supplementation of 5.8 mM MgCl_2_ enhanced the recovery rate in the wound-healing assay, which was inhibited by NIPAL4 siRNA. Pharmacological inhibition experiments showed that the MSK1/GSK3/CREB pathway may be involved in the elevation of HAS2/3 expression, and HA production by MgCl_2_ supplementation. This is the first report showing that Mg^2+^ may be involved in the production of HA in the skin. A reduction of acylceramide, a skin barrier lipid, is reported in NIPAL4-KO mice [3]. We suggest that cosmetic products containing a high concentration of magnesium may be useful to enhance moisturization and wound repair mediated by the elevation of HA and acylceramide production in the skin.

## 4. Materials and Methods

### 4.1. Materials

Goat anti-β-actin and mouse anti-HAS2 antibody were obtained from Santa Cruz Biotechnology (Santa Cruz, CA, USA). Rabbit anti-HAS3 and mouse anti-nucleoporin p62 antibodies were from ProteinTech (Tokyo, Japan) and BD Biosciences (Franklin Lakes, NJ, USA), respectively. Rabbit anti-p-CREB antibody and Proteome Profiler Human Phospho-Kinase Array Kit were from R&D Systems (Minneapolis, MN, USA). KMG-20-AM was from Fujifilm Wako Pure Chemical (Osaka, Japan). Hyaluronan Quantification Kit was from PG Research (Tokyo, Japan). The siRNA against NIPAL4 was from Sigma-Aldrich (St. Louis, MO, USA). The GLuc-ON Promoter Reporter vectors for human HAS2 and HAS3 were from GeneCopoeia (Rockville, MD, USA). All other reagents were of the highest grade of purity available.

### 4.2. Cell Cultures

HaCaT cells, an immortalized non-tumorigenic cells derived from human keratinocyte [30], were grown in Dulbecco’s modified Eagle’s medium (DMEM, Sigma-Aldrich) as described previously [31]. One day before the experiments, cells were transferred to fetal bovine serum (FBS)-free medium. Mg^2+^-free medium was prepared according to the composition of normal DMEM without Mg^2+^.

### 4.3. Transfection of siRNA and Reporter Assay

The siRNAs against human NIPAL4 were transfected into the cells using ScreenFect A (Fujifilm Wako Pure chemical). Mission siRNA Universal Negative Control (Sigma-Aldrich) was used as a negative control. Cells were collected after three days of transfection. The GLuc-ON Promoter Reporter vectors for human HAS2 and HAS3 were transfected into the cells using HilyMax (Dojindo Laboratories, Kumamoto, Japan). The mutants of putative CREB-binding sites of promoter region in HAS2/3 were constructed using a KOD-Plus Mutagenesis kit (Toyobo, Osaka, Japan) and primer pairs as described in Table 1. Transfection efficiency was corrected by secreted alkaline phosphatase (SEAP) reporter gene assay. The activities of secreted luciferase and SEAP were measured using a Ready-To-Glow Dual Secreted Reporter Assay kit (Takara Bio, Shiga, Japan).

### 4.4. Isolation of Total RNA and Quantitative Real-Time PCR

Total RNA was extracted using TRI reagent (Molecular Research Center, Cincinnati, OH, USA). Reverse transcription was carried out using ReverTraAce qPCR RT Kit (Toyobo). Quantitative real-time PCR was performed with Eco Real-Time PCR system (AS One, Osaka, Japan) using Thunderbird SYBR qPCR Mix (Toyobo). The primer pairs used for PCR are listed in Table 2. The threshold cycle (Ct) for each PCR product was calculated with the instrument’s software. The relative change in mRNA expression was calculated as a ratio (R) according to the equation R = 2^ΔCt(treatment)−^^ΔCt(Control)^.

### 4.5. SDS-Polyacrylamide Gel Electrophoresis and Western Blotting

Cells were scraped into cold phosphate buffered saline and precipitated by centrifugation. They were lysed in a RIPA buffer containing 150 mM NaCl, 0.5 mM EDTA, 1% Triton X-100, 0.1% SDS, 50 mM Tris-HCl (pH 8.0), a protease inhibitor cocktail (Sigma-Aldrich), and sonicated for 20 s. After centrifugation at 6000× *g* for 5 min, the supernatants were collected and used as cell lysates which including membrane and cytoplasmic proteins. SDS-polyacrylamide gel electrophoresis and Western blotting were performed as described previously [31].

### 4.6. Measurement of [Mg^2+^]_i_

Cells were seeded at densities of 5 × 10^3^ on a 96 well plate. After three days, the cells were cultured in the FBS-free medium containing 0, 0.8, and 5.8 mM MgCl_2_ for 24 h. The cells were incubated with KMG-20-AM (Fujifilm Wako Pure Chemical) for 30 min at 37 °C. After washing with dye-free Hank’s balanced salt solution two times, the plate was set on a fluorescence microplate reader (Infinite F200 PRO, Tecan, Mannedorf, Switzerland). The fluorescence intensity of KMG-20 was monitored at 430 nm/535 nm and 485 nm/535 nm. [Mg^2+^]_i_ was calculated from the 485 nm/430 nm ratio, which can compensate cell number and dye leakage, and represented relative to the value of control cells (0.8 mM MgCl_2_).

### 4.7. Measurement of HA Content

Cells were cultured on 6 well plates and the medium was replaced fresh one before one day of assay. HA content was measured using a Hyaluronan Quantification Kit (PG Research, Kodaira, Tokyo, Japan).

### 4.8. Wound-Healing Assay

Cells were cultured on 6 well plates and a scratch was generated with a 200-μL pipette tip. Then, the cells were incubated in 0, 0.8, and 5.8 mM MgCl_2_-containing media. In order to avoid the effect of cell proliferation, the concentration of FBS was reduced to 0.5%. The images of cells were taken using Olympus inverted microscope CKX53 equipped with TrueChromeII Plus at 0 and 12 h after scratching. Cell migration was calculated with the area of the initial wound using ImageJ software.

### 4.9. Statistical Analysis

Results are presented as means ± S.E.M. Differences between groups were analyzed using one-way analysis of variance, and corrections for multiple comparison were made using Tukey’s multiple comparison test. Comparisons between two groups were made using Student’s *t* test. Statistical analyses were performed using KaleidaGraph version 4.5.1 software (Synergy Software, Reading, PA, USA). Significant differences were assumed at *p* < 0.05.

## Figures and Tables

**Table 1:** Primer pairs for mutation.

Name	Direction	Sequence
HAS2m	Forward	5′-TAGCTGCAGCTCAGAAACTTTTGAGTT-3′
	Reverse	5′-AGTGTCAAAGCCTTTCTCAT-3′
HAS3m	Forward	5′-ATGCCACCGAGGCGGGGCGCCAGCG-3′
	Reverse	5′-GCCGGAGGCGGCGCCCACCAG-3′

**Table 2:** Primer pairs for real-time PCR.

Name	Direction	Sequence
HAS2	Forward	5′-CTGGGCTATGCAACAAAATACA-3′
	Reverse	5′-TTCTCGGAAGTAGGACTTGCTC-3′
HAS3	Forward	5′-AGAAGTTCCTAGGCAGCAAGTG-3′
	Reverse	5′-GGAGGTACTTAGTGGGGGTCTC-3′
NIPAL4	Forward	5′-ACATGCTCCTGAGGAAGAGAAG-3′
	Reverse	5′-GCAATGACAAAGATGAGGATGA-3′
β-Actin	Forward	5′-CCTGAGGCACTCTTCCAGCCTT-3′
	Reverse	5′-TGCGGATGTCCACGTCACACTTC-3′

## Data Availability

Not applicable.

## Supplementary Materials

The following are available online at https://www.mdpi.com/article/10.3390/ijms23010071/s1.
