# Supplementary material for: Elevation of Hyaluronan Synthase by Magnesium Supplementation Mediated through the Activation of GSK3 and CREB in Human Keratinocyte-Derived HaCaT Cells
Source: Int J Mol Sci. 2021 Dec 22;23(1):71. doi: 10.3390/ijms23010071 (PMC8744730; doi:10.3390/ijms23010071)
Supplement: Supplementary file 1 [file ijms-23-00071-s001.zip › ijms-1526156-supplementary.pdf]

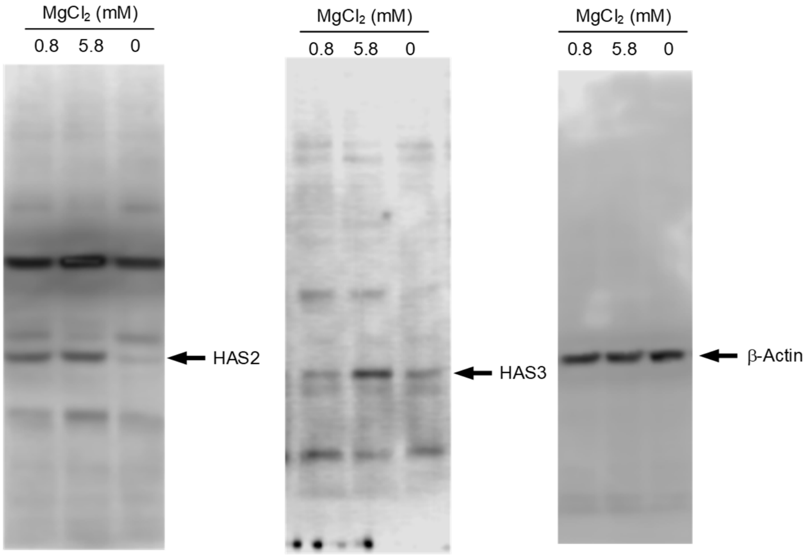

Figure S1. Original Images of figure 1C

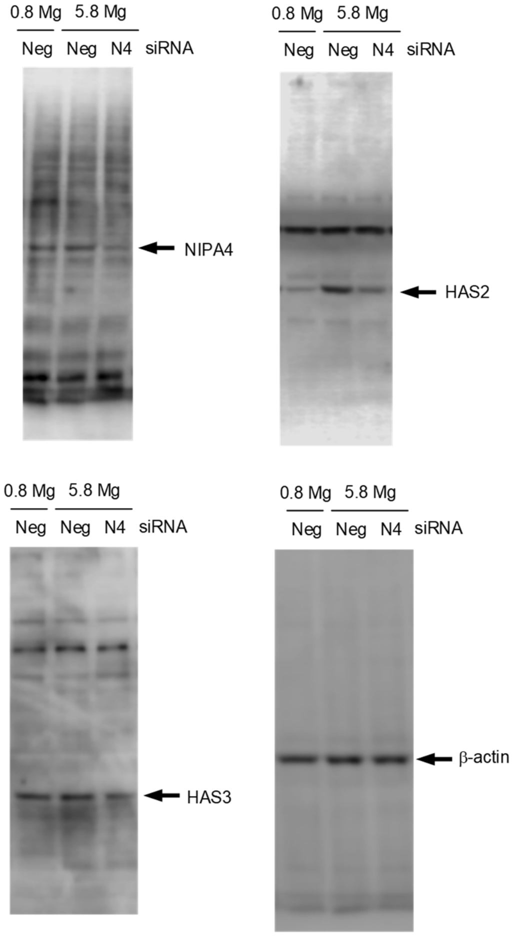

Figure S2. Original Images of figure 2C

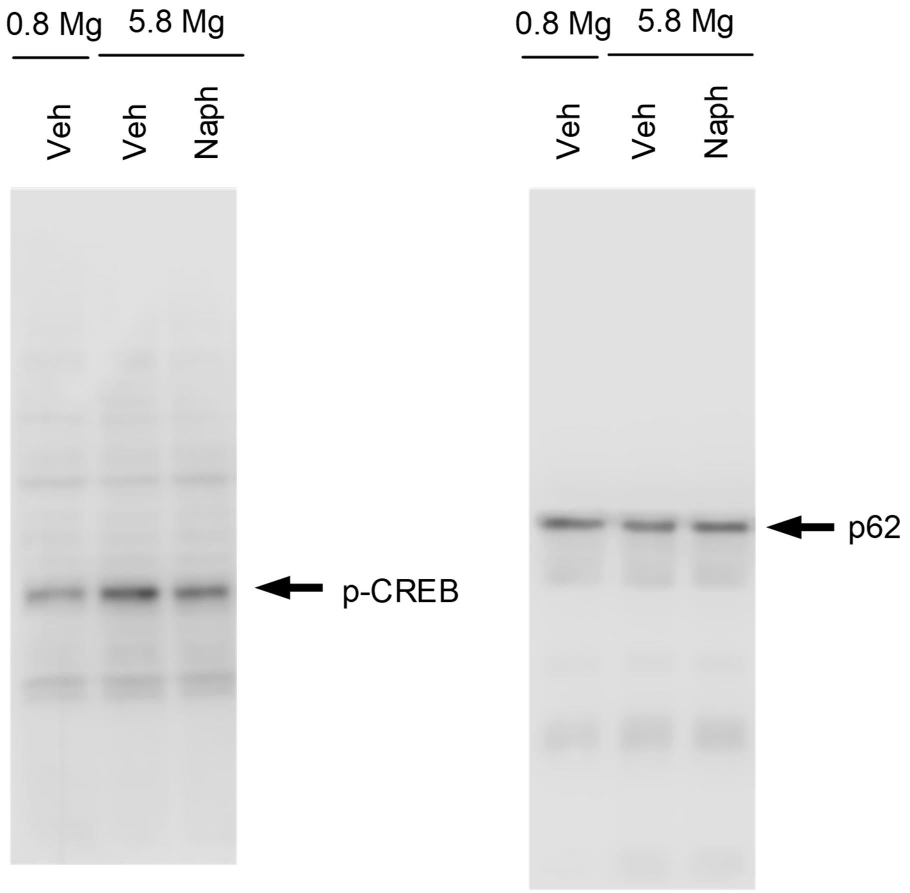

Figure S3. Original Images of figure 8A
